# Police Officer Perceptions of Non-consensual Dissemination of Intimate Images

**DOI:** 10.3389/fpsyg.2020.02148

**Published:** 2020-09-03

**Authors:** Liza Zvi, Mally Shechory-Bitton

**Affiliations:** Department of Criminology, Ariel University, Ariel, Israel

**Keywords:** police officers, non-consensual dissemination of intimate images, forensic judgments, technology facilitated sexual violence, online victimization

## Abstract

Non-consensual dissemination of intimate images (NCII) is a major concern in many countries. The increase in the number of NCII cases and awareness of its adverse effects on victims has raised public awareness, with many states enacting legal and non-legal measures to combat this new type of violence. Yet, despite recent legislation, there is a reason to suspect that the majority of NCII cases remain unreported. Thus, research is needed on law enforcement perceptions of victims and identification of victim-blaming attitudes and factors that might affect legal decision-making. The present study addressed this issue by focusing on Israeli police officer perceptions of NCII victims and offenders: 145 police officers and 160 students, who served as a control group, were presented with a scenario depicting an NCII offense in which the stolen intimate material was either self-generated by the victim (selfies) or stealth-taken by the victim’s ex-boyfriend. In both cases, the stolen images were disseminated by the ex-boyfriend without the victim’s permission. The findings indicated victim-blaming attitudes toward NCII victims within law enforcement as well as an effect of the source of stolen images. Although officers perceived NCII as criminal and the offender as highly culpable and punishable, they engaged in victim-blaming. This was especially the case for the self-taken scenario, which elicited negative feelings and less empathy toward the victim. The relevance of emotions in legal contexts is emphasized in light of their contribution to the participants’ punitive judgments. Victim-blaming in NCII offenses and its implications are discussed, and suggestions are made for how to reduce negative and victim-blaming attitudes among law enforcement.

## Introduction

Police officers are the “gatekeepers” of the criminal justice system and are usually the first to come into contact with crime victims. As such, they exert major influence on both the victim and the case (Hine and Murphy, 2019). Police work is discretionary by nature, and officer perception of cases, victims, and offenders may impact decisions, such as which cases are worth pursuing, amount of investigative efforts a case will receive, and how to conduct the investigation. Even though neutrality and objectivity of law enforcement is emphasized, real-life circumstances show that reality is more complex, especially with respect to sexual offenses. As most sexual offenses are non-stranger assaults, tangible evidence is often lacking, leaving room for different competing narratives as to what really happened and to personal inferences and bias (Willmott et al., 2018).

Research on forensic biases among police officers is relatively limited. However, it shows that officers hold rape myths, specifically those related to victim credibility and blame-worthiness (e.g., Page, 2007, 2010; Goodman-Delahunty and Graham, 2011; Sleath and Bull, 2012; Bull and Milne, 2020) and that their perception of victims might be biased by irrelevant factors (e.g., victims’ clothing, prior relationship with the assailant, and alcohol intoxication; for a review, see Sleath and Bull, 2017). The way the victim is perceived may subsequently impact the way the victim is treated, as well as officer decision-making in the case. It has been well-established that the progress of cases through the criminal justice system reflects a highly selective process of elimination, also referred to as attrition, and that this is especially true for sexual assault cases (Soulliere, 2005). Statistics show that rape cases are characterized by high attrition rates, which are greatest at the investigative stage (Parratt and Pina, 2017; Sleath and Bull, 2017). Moreover, rape victims who approached the police have reported low levels of satisfaction, feelings of not being taken seriously and being poorly or negatively treated (Jordan, 2001, 2004; Hammond et al., 2017). This further raises concern as to a possible impact of officer perceptions and judgments.

Research on law enforcement perceptions has focused on police officer perceptions of rape (see, for example, Sleath and Bull, 2012; Shechory Bitton and Jaeger, 2019). However, law enforcement perceptions of other types of sex offenses, such as cyber-sex crimes, have rarely been studied. With the advent of the Internet and communication technologies, a new type of sexual violence has emerged – technology-facilitated sexual violence (TFSV; Henry et al., 2018a). This form of criminal violence is now the focus of a great deal of research. Indeed, crimes such as cyber fraud and hacking have drawn considerable attention. However, the research on TFSV has tended to focus on minors and much less so on adults (Henry and Powell, 2016, 2018; Henry et al., 2018a; Zvi and Shechory-Bitton, 2020).

TFSV includes a range of sexually aggressive behaviors perpetrated using digital technologies, among them non-consensual dissemination of intimate images (NCII). NCII is a form of image-based sexual abuse that has gained an increasing amount of public and legal attention in recent years (McGlynn and Rackley, 2017). NCII may be perpetrated by current or past intimate partners, friends and acquaintances, family members, or persons unknown to the victim for various reasons, including revenge, blackmail or coercion, sexual gratification, fun, and social status (Henry et al., 2019). Source of the disseminated material may vary. In some instances, victims have taken their own intimate images (“selfies”) or consented to someone else taking their images but never consented to their distribution. In other cases, intimate images of the victims were stealth-taken, without their knowledge (Henry and Powell, 2015, 2016). The distribution of intimate material is not only easily facilitated, primarily using social media platforms (e.g., Facebook, Instagram, and Snapchat), but also *via* smartphones and dedicated websites (Franklin, 2014; Sherlock, 2016; McGlynn et al., 2017). It was estimated that there are around 3,000 websites dedicated to NCII (e.g., the notorious “Is anyone up?” website, which was shut down; McGlynn et al., 2017).

Once disseminated, the intimate material will most likely exist forever in digital memory, exposing victims to permanent reputational and social harm and ongoing victimization (Dodge, 2019). Victims suffer from serious mental and physical consequences, similar to those seen in conventional sexual offenses, including depression, profound fears and anxiety, post-traumatic stress disorder, and overuse of drugs and alcohol as means of coping. Shame, low self-esteem, and loss of trust in others are common and might be further exacerbated if friends, family, colleagues, and employers are exposed to the victim’s intimate photos (Citron and Franks, 2014; Kamal and Newman, 2016; Bates, 2017).

The rise in NCII cases and awareness of their detrimental effects on victims has made this relatively new form of violence a major concern in many countries, resulting in a search for solutions. Over the past 6 years, many countries have made criminal law reforms or enacted legal and non-legal measures in order to combat NCII (Henry et al., 2018a). Media coverage of high-profile cases has also contributed to increased awareness of the problem (e.g., famous Hollywood actresses whose intimate photos were stolen and disseminated, local cases of large-scale dissemination of intimate materials of anonymous women, or cases involving local celebrities). Yet, being a relatively new phenomenon, only a few studies have addressed the issue of perceptions of NCII (Bothamley and Tully, 2018; Scott and Gavin, 2018; Zvi and Shechory-Bitton, 2020). To our knowledge, only one study has addressed the issue of police officers, examining 783 UK police officers and their knowledge of and experience with NCII cases (Bond and Tyrrell, 2018). It was found that the participants had limited understanding of these sorts of crimes and lacked confidence in investigating them and in responding to victims. The authors recommended improved police training and resources. Another study looked at the experiences of 52 Australian legal and policy experts, domestic and sexual violence advocates, industry representatives, police, and academics in relation to policing and image-based sexual abuse (Henry et al., 2018b). Drawing on interviews with these stakeholders, the authors identified five key barriers to law enforcement responses to image-based sexual abuse in Australia: inconsistent laws, a lack of resources, evidentiary limitations, jurisdictional boundaries, and victim-blaming or a harm minimization attitude. It was concluded that there is much to be done within the criminal justice system to improve responsiveness.

Thus, the current research is focused on expanding the limited knowledge of police perceptions of NCII by evaluating Israeli police officer perceptions toward NCII victims and offenders. In Israel, legislation from 2014 prohibits the distribution of intimate images, films, or tapes of another person without their consent. This legislation is included in laws against sexual harassment, thus recognizing NCII as an offense of a sexual nature with punitive consequences like that for sexual harassment – up to 5 years in prison.

Despite worldwide legislation, criticism has been raised by scholars and legal professionals that NCII laws are restricted in scope and sometimes offer only limited redress (McGlynn et al., 2017; Lageson et al., 2018). One major difficulty is related to an ambiguity with respect to victim consent, which in cases of selfies or material generated with victim consent may be open to varied interpretation (Citron and Franks, 2014). Specifically, in the case of an individual who agreed to be privately photographed/video recorded in intimate circumstances, this may be construed as consent to share said images/video (Citron and Franks, 2014). Furthermore, victim precipitation theory (e.g., Schafer, 1968, 1977) posits that victims contribute to some degree to their victimization, which suggests that higher levels of victim-blaming toward victims whose images were self-taken might occur. Although unconsented distribution of intimate material of any source constitutes an offense under the Israeli law, cases where the victim’s material was self-generated or generated with their consent may be perceived and regarded differently. A recent study of 250 Israeli students corroborated this premise by showing biased perceptions toward a NCII victim whose stolen intimate images were self-taken (Zvi and Shechory-Bitton, 2020). In the current study, officer perceptions were assessed with reference to the different circumstances of NCII as well as in comparison to student perceptions.

Police officers are expected to possess greater awareness of NCII and combating NCII than the non-law enforcement population. They are also expected to have greater familiarity with recent legislation along with knowledge that unconsented distribution of intimate images, both self-taken and stealth-taken (i.e., taken without the victim’s knowledge), constitutes a criminal act. Thus, officers are expected to show more objective and impartial judgments regarding NCII in comparison to other persons without legal knowledge and experience. Yet still, rape research continues to show that police officers engage in victim-blaming (e.g., Venema, 2016). Based on the above, we hypothesized that police officers will engage in victim-blaming but at lower levels than students. The police officers were also expected to apply more objective reasoning regarding offender culpability and appropriate punishment. Another aim was to evaluate gender differences in blame attributions and judgments. Studies have consistently shown sex differences in men and women’s blame attributions and judgments of victims and offenders (e.g., Shechory-Bitton and Zvi, 2015; Fido et al., 2018) and that, with respect to sexual assault, men tend to attribute more blame and responsibility than women to victims (e.g., Ferrão and Gonçalves, 2015; Shechory Bitton and Jaeger, 2019). It was thus hypothesized that male participants would be more blaming toward NCII victims relative to female participants.

In order to test the hypotheses, we used a scenario of non-consensual distribution of intimate photographs of a 30-year-old female by her ex-boyfriend. The scenario contained either images taken by the victim or images that were stealth-taken by the victim’s ex. To avoid any misunderstanding regarding consent, both scenarios depicted circumstances, where the victim’s photos were obtained without their consent. In the selfie scenario, the victim did not willingly share the private images with the offender, and it was stressed that she was photographing herself for private use only, with no intention of sharing her intimate material with anyone. After reading the scenario, the participants were asked to rate their perceptions of blame toward the victim and offender and deserved punishment for the offender. Participants were also asked to indicate their feelings toward the victim and offender. Previous research suggests that feelings may serve as indirect indicators of blame attributions. It was shown that levels of blame and responsibility attributed to victims of sexual assault were related to participant levels of empathy (Deitz et al., 1982, 1984; Smith and Frieze, 2003; Miller et al., 2011; Ferrão and Gonçalves, 2015). As for offenders, responsibility was related to participant reported anger toward the offender (Keltner et al., 1993; Bright and Goodman-Delahunty, 2006). Thus, we assessed participant positive and negative feelings toward the victim and offender, in addition to a direct measurement of their blame attributions.

## Materials and Methods

### Participants

Participants were 145 police officers and 160 students from various disciplines at a large university in Israel. There were 66 male police officers (45.5%) and 79 female police officers (54.5%), with 79 male students (49.4%) and 81 female students (50.6%). Participant ages ranged between 19 and 60 years, with a mean of 27.20 years (*SD* = 6.42; range for police officers: 20–60 years, range for students: 19–37 years). Police officers were older than students [*M* = 29.69, *SD* = 8.19 vs. *M* = 24.93, *SD* = 2.72, *t*(172.65) = 6.67, *p* < 0.001], and males were slightly older than females [*M* = 28.33, *SD* = 6.16 vs. *M* = 26.16, *SD* = 6.50, *t*(303) = 2.99, *p* = 0.003]. Most participants were Jewish (*N* = 282, 92.5%). About half of the participants were single (*N* = 153, 50.2%), while the others were mostly married or living with a companion (*N* = 146, 47.9%), with no group or gender difference. Most did not have children (*N* = 230, 75.4%), to a greater extent among students (*N* = 134, 83.8%) than among police officers (*N* = 96, 66.2%; *Z* = 3.55, *p* < 0.001). No difference was found in age and family status according to group (self-taken/stealth-taken).

### Instruments

*Demographic Questionnaire*: This measure included questions on age, sex, and family status.

#### Vignettes

Two vignettes described scenarios of non-consensual distribution of intimate photographs of a 30 year-old female by her ex-boyfriend. The cases were completely identical in all aspects aside from the photograph source. In one scenario, the photographs were taken by the victim herself, whereas in the other they were stealth-taken by the victim’s ex. In the selfie scenario it was emphasized that the photographs taken by the victim were for her personal use only. Each participant was presented with one of the two versions of NCII. Below are the vignettes:

One morning, Osnat, a young 30-year-old, received a phone call from a female friend saying that intimate photographs of Osnat were circulating on social media. At first, Osnat thought that her friend was pranking her. However, other phone calls from friends and family members made her realize that it was real. She felt that her life was destroyed. During summer vacation, she took some naked pictures of herself, but they were only for her own personal use. She remembered having kept these pictures in her secure possession and could not understand how the pictures ended up on the Internet or who distributed them. Osnat went to the police and asked them to investigate. The investigation revealed that it was Yariv, her ex-boyfriend, who secretly made himself a copy of the pictures and decided to distribute them.
One morning, Osnat, a young 30-year-old, received a phone call from a female friend saying that intimate photographs of Osnat were circulating on social media. At first, Osnat thought that her friend was pranking her. However, other phone calls from friends and family members made her realize that it was real. She felt that her life was destroyed. She was never photographed naked and could not conceive of how pictures of her ended up on the Internet or who distributed them. Osnat went to the police and asked them to investigate. The investigation revealed that it was Yariv, her ex-boyfriend, who had secretly taken her pictures and decided to distribute them.

#### Victim and Offender Blame Scale

Three questions were used to assess blame attributions toward the offender and victim: (1) the offender is entirely to blame; (2) blame is shared by both parties (joint blame); and (3) the victim is entirely to blame. Responses to each item were given on a scale of 1 (very little agreement) to 5 (very high agreement). The three blaming items were taken from previous research on perceptions of criminal cases (e.g., Shechory-Bitton and Zvi, 2015, 2016, 2019).

#### Feelings Toward the Victim and Offender

As feelings toward the victim and offender may serve as indirect indicators of blame, participants were asked to report their negative and positive feelings toward victim and offender using the following two items: “*Please indicate to what extent you feel the following feelings toward Osnat*” and “*Please indicate to what extent you feel the following feelings toward Yariv*.” Two items were used to asses positive, empathetic feelings (feeling sorry for and compassion toward the target) and two were used to assess negative feelings (feeling anger and contempt toward the target). For each item, responses were given on a five-point scale 1 (slightly) to 5 (considerably). The two positive feelings correlated at *r*_s_ = 0.41 (*p* < 0.001) regarding the victim and at *r*_s_ = 0.50 (*p* < 0.001) regarding the offender. The two negative feelings correlated at *r*_s_ = 0.55 (*p* < 0.001) regarding the victim and at *r*_s_ = 0.48 (*p* < 0.001) regarding the offender. Thus, four variables were composed of the item means: positive feelings and negative feelings toward the victim and positive feelings and negative feelings toward the offender.

#### Offender Punishment Scale

The participants were asked about the proper punishment using the following three items: (1) the offender should be brought to trial; (2) the offender should be given minimum punishment; and (3) the offender should be given maximum punishment. Responses to each item were given on a scale of 1 (very little agreement) to 5 (very high agreement).

### Procedure

Undergraduate students were randomly approached in the university and were asked to voluntarily participate in the study. Police officers also voluntarily participated. The main obstacle to gathering data derived from the difficulty of locating police officers. In an attempt to overcome this problem to the fullest degree possible, locating police officer participants for the study was achieved through a snowball process (see also Javaid, 2017; Shechory Bitton and Jaeger, 2019), in which criminology students, also volunteering in police units and acquainted with police officers, asked officers to participate and recruit other police officers. The police officers in the study are in “field” roles (investigations, reconnaissance, etc.). As such, they deal with a variety of events, including events related to sexual delinquency as well as NCII cases. Recruiting police officers to participate granted the students with course credit. The participants were handed the questionnaires and were asked to return them to the volunteering students upon completion. Police officers received the questionnaires in their units and returned them in sealed envelopes. All the participants were assured that their responses would be completely anonymous and confidential and used only for research purposes. They were also told that they could refuse to participate in the study without penalty and could withdraw their participation at any stage. After providing informed consent, they were given one of the two research scenarios, followed by the research questionnaire. The questionnaires, procedures, informed consent form, and instructions were reviewed and approved by the Ethics Committee of the university. A total of 340 questionnaires were distributed. Of these, 315 were returned to the researchers. Ten questionnaires were excluded for not having been fully completed. A total of 305 questionnaires were utilized. The participants were randomly assigned to the two research conditions: self-taken (*N* = 154, 50.5%) and stealth-taken (*N* = 151, 49.5%). Each sub-group in the 2 × 2 × 2 design (police officers/students × gender × research condition) included 30–41 participants.

### Data Analysis

Data were analyzed with SPSS ver. 26. Descriptive statistics was first used with the study variables. As the variables representing blame, punishment, positive feelings, and negative feelings were non-normally distributed, they were either log transformed (for positive values of skewness) or exponentially transformed (for negative values of skewness). Thus, skewness values of −2.38–1.62 turned into −1.14–1.07 (*SE* = 0.14). Differences in blame, punishment, positive feelings, and negative feelings by group (students/police officers), research condition (self-taken/stealth-taken), and the respondent’s gender were calculated with three way analyses of variance (2 × 2 × 2). Multiple hierarchical regression models were calculated to assess the contribution of the main study variables: blame and feelings, to the punishment scores. The first step included group, research condition, and gender, and the second step included the two variables of blame (victim’s and shared blame) and the four positive and negative feelings. The regression models were added alongside the ANOVAs in order to assess the relative contribution of blame and feelings to the punishment scores.

A power analysis for the three-way ANOVAs (2 × 2 × 2) revealed that with a low-moderate effect size of *f* = 0.16 (equals to *η*^2^ = 0.04), and *α* = 0.05, the sample size of *N* = 305 yields a power of 0.80. For a regression model with nine predictors, this sample size, with a low-moderate effect size of *f*^2^ = 0.10 (equals to *R*^2^ = 0.10), and *α* = 0.05, the sample size of *N* = 305 yields a power of 0.98 (Power analyses were conducted with G^*^Power 3, Faul et al., 2007, 2009). According to Lenhard and Lenhard (2016), an effect size of *η*^2^ = 0.01 was considered small, an effect size of *η*^2^ = 0.09 was considered moderate, and an effect size of *η*^2^ = 0.25 was considered large.

## Results

### Blame and Punishment

Most participants thought that the offender was highly blameworthy (*N* = 235, 77.0%) or blameworthy (*N* = 52, 17.0%), and only a few ascribed to him moderate or little blame (*N* = 18, 6.0%). Likewise, most participants were highly convinced (*N* = 251, 82.3%) or convinced (*N* = 36, 11.8%) that the offender should be brought to trial, and only some were hesitant in that respect (*N* = 18, 5.9%). Greater variation was found in the responses pertaining to the extent to which the victim was perceived as blameworthy or the extent to which blame was perceived as shared by both parties. About 60% of the participants ascribed very little (*N* = 189, 62.0%) or little blame to the victim (*N* = 39, 12.8%), and most others attributed moderate blame (*N* = 45, 14.8%) to the victim. About 65% of the participants ascribed very little (*N* = 197, 64.6%) or little shared blame to the victim and offender (*N* = 47, 15.4%), and most others attributed victim and offender with moderate blame (*N* = 34, 11.1%). Likewise, variation was found regarding giving the offender maximum or minimum punishment. About 55% of the participants highly agreed (*N* = 173, 56.7%) or agreed (*N* = 56, 18.4%) with maximum punishment, and most others moderately agreed with maximum punishment (*N* = 50, 16.4%). About 60% of the participants highly agreed (*N* = 191, 62.6%) or agreed (*N* = 43, 14.1%) with minimum punishment, and most others moderately agreed with minimum punishment (*N* = 39, 12.8%). Distributions are shown in Table 1. It should be noted that as the four variables in Table 1 are non-normally distributed they were log transformed (victim’s sole blame, victim and offender share blame, and minimum punishment) or exponentially transformed (maximum punishment).

**Table 1:** Raw means and standard deviations for blame and punishment by group, condition, and gender (*N* = 305).

	Total sample	Group		Condition		Gender		Group by condition			
		Students	Police officers	Self-taken	Stealth-taken	Male	Female	Students ‐ self-taken	Students ‐ stealth-taken	Police officers ‐ self-taken	Police officers ‐ stealth-taken
	*M* (*SD*) (*N* = 305)	*M* (*SD*) (*N* = 160)	*M* (*SD*) (*N* = 145)	*M* (*SD*) (*N* = 154)	*M* (*SD*) (*N* = 151)	*M* (*SD*) (*N* = 145)	*M* (*SD*) (*N* = 160)	*M* (*SD*) (*N* = 80)	*M* (*SD*) (*N* = 80)	*M* (*SD*) (*N* = 74)	*M* (*SD*) (*N* = 71)
Victim’s sole blame	1.78 (1.16)	1.76 (1.10)	1.79 (1.22)	2.26 (1.32)	1.28 (0.68)	1.93 (1.22)	1.64 (1.08)	2.35 (1.17)	1.18 (0.61)	2.16 (1.46)	1.41 (0.73)
Victim and offender share blame	1.67 (1.07)	1.64 (1.02)	1.71 (1.14)	2.00 (1.23)	1.34 (0.76)	1.73 (1.09)	1.62 (1.06)	2.04 (1.18)	1.24 (0.6)	1.96 (1.29)	1.45 (0.89)
Maximum punishment	4.18 (1.13)	4.23 (1.08)	4.14 (1.19)	4.05 (1.23)	4.32 (1.02)	4.08 (1.22)	4.28 (1.05)	4.06 (1.25)	4.39 (0.86)	4.04 (1.22)	4.24 (1.16)
Minimum punishment	1.76 (1.16)	1.70 (1.13)	1.82 (1.20)	1.88 (1.23)	1.64 (1.08)	1.79 (1.13)	1.73 (1.19)	1.95 (1.32)	1.45 (0.83)	1.80 (1.12)	1.85 (1.28)

Results show that the general mean for the *Victim’s sole blame* was rather low (1.78 in a range of 1–5). A three-way ANOVA revealed a significant condition difference [*F*(1, 297) = 67.62, *p* < 0.001, *η*^2^ = 0.185] with a moderate effect size, such that the victim’s blame was higher in the self-taken condition than in the stealth-taken condition. A significant gender difference was found as well [*F*(1, 297) = 6.42, *p* = 0.012, *η*^2^ = 0.021], revealing that males tended to blame the victim more than females, with small effect size. The group difference was not significant. Further, the interaction of group by condition was significant [*F*(1, 297) = 7.29, *p* = 0.007, *η*^2^ = 0.024], showing that difference by condition is significant for both students [*F*(1, 297) = 63.18, *p* < 0.001, *η*^2^ = 0.175] and police officers [*F*(1, 297) = 14.45, *p* < 0.001, *η*^2^ = 0.046]. Even so, the magnitude of the difference is greater for students, as the effect size is moderate compared with the other one that is rather small. That is, students tended to attribute greater blame to the victim in the self-taken condition than in the stealth-taken condition, to a greater extent than did the police officers. Other interactions were not significant.

The general mean for the *Victim and offender shared blame* was rather low as well (1.67 in a range of 1–5). The only significant difference found was a condition difference [*F*(1, 297) = 31.51, *p* < 0.001, *η*^2^ = 0.096], with a moderate effect size, so that victim and offender shared blame was higher when the victim’s photos were self-taken than when they were not.

The general mean for supporting *Maximum punishment* was high (4.18 in a range of 1–5), with no significant differences by group, condition, or gender.

The general mean for supporting *Minimum punishment* was low (1.76 in a range of 1–5). A significant condition difference was found [*F*(1, 297) = 4.32, *p* = 0.038, *η*^2^ = 0.015], with a low effect size, such that minimum punishment was more highly supported when the victim’s photos were self-taken than when they were not. Further, the interaction of group by condition was significant [*F*(1, 297) = 4.39, *p* = 0.037, *η*^2^ = 0.015], showing that difference by condition is significant for students [*F*(1, 297) = 9.24, *p* = 0.003, *η*^2^ = 0.031], with a low effect size, but not significant for police officers [*F*(1, 297) = 0.01, *p* = 0.987, *η*^2^ = 0.001]. That is, students tended to support minimum punishment more in the self-taken condition than in the stealth-taken condition, but no difference was found among the police officers.

### Feelings Toward the Victim and the Offender

The results (Table 2) show that the general mean for positive feelings toward the victim was high (4.37 in a range of 1–5). A three-way ANOVA revealed a significant condition difference [*F*(1, 297) = 7.04, *p* = 0.008, *η*^2^ = 0.023], with a low effect size, such that positive feelings were lower toward the victim whose images were self-generated. A significant gender difference was found as well [*F*(1, 297) = 27.01, *p* < 0.001, *η*^2^ = 0.084], with quite a moderate effect size, revealing that females felt more positively toward the victim than males. The group difference and all interactions were not significant.

**Table 2:** Raw means and standard deviations for feelings toward the victim and the offender, by group, condition, and gender (*N* = 305).

	Total sample	Group		Condition		Gender		Group by condition			
		Students	Police officers	Self-taken	Stealth-taken	Male	Female	Students ‐ self-taken	Students ‐ stealth-taken	Police officers ‐ self-taken	Police officers ‐ stealth-taken
	*M* (*SD*) (*N* = 305)	*M* (*SD*) (*N* = 160)	*M* (*SD*) (*N* = 145)	*M* (*SD*) (*N* = 154)	*M* (*SD*) (*N* = 151)	*M* (*SD*) (*N* = 145)	*M* (*SD*) (*N* = 160)	*M* (*SD*) (*N* = 80)	*M* (*SD*) (*N* = 80)	*M* (*SD*) (*N* = 74)	*M* (*SD*) (*N* = 71)
Victim‐ positive feelings	4.37 (0.82)	4.47 (0.71)	4.27 (0.92)	4.25 (0.87)	4.50 (0.76)	4.19 (0.87)	4.54 (0.74)	4.39 (0.79)	4.55 (0.61)	4.10 (0.92)	4.44 (0.89)
Victim-negative feelings	1.80 (1.06)	1.70 (0.98)	1.91 (1.14)	2.09 (1.13)	1.50 (0.90)	1.85 (1.05)	1.75 (1.08)	2.08 (1.07)	1.33 (0.72)	2.11 (1.20)	1.69 (1.04)
Offender-positive feelings	1.65 (1.04)	1.72 (1.10)	1.57 (0.96)	1.66 (1.05)	1.64 (1.03)	1.66 (1.03)	1.64 (1.04)	1.74 (1.15)	1.71 (1.06)	1.56 (0.93)	1.57 (0.99)
Offender-negative feelings	4.57 (0.81)	4.66 (0.70)	4.47 (0.91)	4.56 (0.83)	4.57 (0.80)	4.43 (0.88)	4.70 (0.74)	4.66 (0.72)	4.66 (0.69)	4.47 (0.93)	4.47 (0.90)

Negative feelings toward the victim were rather low (mean of 1.80 in a range of 1–5). A significant condition difference was found [*F*(1, 297) = 30.66, *p* < 0.001, *η*^2^ = 0.094], with a moderate effect size, such that negative feelings toward the victim were higher when the photos were self-taken than when they were stealth-taken. The interaction of group by condition was significant [*F*(1, 297) = 4.22, *p* = 0.041, *η*^2^ = 0.014], revealing that the difference by condition was significant for both students [*F*(1, 297) = 30.51, *p* < 0.001, *η*^2^ = 0.094] and police officers [*F*(1, 297) = 5.74, *p* = 0.017, *η*^2^ = 0.019], yet the magnitude of the difference was greater for students, as the effect size is moderate compared with the other one that is rather small. In addition, the group difference was significant for the self-taken condition [*F*(1, 297) = 6.53, *p* = 0.011, *η*^2^ = 0.022] but not for the stealth-taken condition [*F*(1, 297) = 0.11, *p* = 0.740, *η*^2^ = 0.001], showing that police officers reported greater negative feelings toward the victim than students in the case of stealth-taken photos, but not in the case of a self-taken photos, although both effect sizes are small.

Positive feelings toward the offender were low (mean of 1.65 in a range of 1–5) and did not differ by group, condition, or gender. Negative feelings toward the offender were high (mean of 4.58 in a range of 1–5). A significant group difference was found [*F*(1, 297) = 4.94, *p* = 0.027, *η*^2^ = 0.016], with a low effect size, with students reporting greater negative feelings toward the offender than police officers. A significant gender difference was found as well [*F*(1, 297) = 14.15, *p* < 0.001, *η*^2^ = 0.045], with a low to moderate effect size, such that negative feelings toward the offender were higher among females than males.

Two multiple hierarchical regression models were calculated to assess the contribution of blame and feelings to the punishment scores. The first step included group (1-students, 0-police officers), condition (1-self-taken, 0-stealth-taken), and gender (1-male, 0-female), and the second step included the two variables of blame (victim’s and shared blame) and the four positive and negative feelings. Collinearity diagnostics revealed a maximum VIF value of 2.20 for the regression models and a maximum condition index of 10.99, and thus not pointing to a multicollinearity problem. Results in Table 3 reveal that both regression models are significant with moderate levels of explained variance: 11% of the variance in maximum punishment and 10% of the variance in minimum punishment were explained in them. In both cases, beyond group, condition, and gender, positive feelings toward the victim and negative feelings toward the offender were found significant in opposite directions. That is, higher positive feelings toward the victim and higher negative feelings toward the offender were related with a greater endorsement of maximum punishment. Similarly, lower positive feelings toward the victim and lower negative feelings toward the offender were related with a greater endorsement of minimum punishment. In addition, along the same lines, lower positive feelings toward the offender and a perception of greater shared blame by victim and offender were both related with a greater endorsement of maximum punishment.

**Table 3:** Multiple regressions for minimum and maximum punishment by blame and feelings (*N* = 305).

	Maximum punishment				Minimum punishment			
	*B*	*SE*	*β*	Δadj.*R*^2^	*B*	*SE*	*β*	Δadj.*R*^2^
Step 1				0.01				0.01
Group	1.99	6.79	0.02		−0.06	0.06	−0.05	
Condition	−12.13	6.78	−0.10		0.11	0.06	0.10	
Gender	−9.18	6.79	−0.08		0.05	0.06	0.05	
Step 2				0.10^***^				0.09^***^
Group	−1.86	6.54	−0.02		−0.02	0.06	−0.02	
Condition	−7.52	7.20	−0.06		0.02	0.07	0.02	
Gender	−0.06	6.73	−0.01		−0.04	0.06	−0.03	
Victim’s sole blame	7.47	8.63	0.07		0.02	0.08	0.02	
Victim and offender share blame	−17.94	8.59	−0.16^*^		0.10	0.08	0.09	
Victim‐ positive feelings	0.15	0.07	0.13^*^		−0.01	0.01	−0.13^*^	
Victim-negative feelings	0.43	7.54	0.01		0.11	0.07	0.10	
Offender-positive feelings	−14.20	6.61	−0.12^*^		0.08	0.06	0.08	
Offender-negative feelings	0.26	0.07	0.21^***^		−0.01	0.01	−0.16^**^	
Total model ‐ Adj.*R*^2^	*F*(9, 295) = 5.30, *p* < 0.001			0.11^***^	*F*(9, 295) = 4.55, *p* < 0.001			0.10^***^

* *p* < 0.05;

** *p* < 0.01;

*** *p* < 0.001.

## Discussion

The findings indicate bias reflected in differing blame attributions (depending on the source of misappropriated images) among police officers and students, although the biases were found to be stronger among students. A similar tendency was also exhibited in participant attitudes toward the need to punish the offender and the severity of the punishment. Although both students and police officers agreed that the offender should be severely punished for his actions, in cases where the victim photographed herself the participants were more forgiving and preferred to ease the offender’s sentence.

The participants reported feelings further showed bias resulting from the source of stolen images. Although, in general, high levels of empathy toward the victim were reported, empathy was significantly less and negative feelings more prominent toward the victim whose images were self-taken. These findings are significant in light of the effect that feelings have on sentencing judgments. In fact, it was found that participant feelings more than their blame attributions predicted their punishment preferences. Along with previous research, this suggests that feelings may serve as indirect indicators of blame. Specifically, participant-reported anger toward offenders was related to their perceptions of offender responsibility (Keltner et al., 1993; Bright and Goodman-Delahunty, 2006). Likewise, participant level of empathy was related to levels of blame and responsibility attributed to victims (Deitz et al., 1982, 1984; Smith and Frieze, 2003; Miller et al., 2011; Ferrão and Gonçalves, 2015). In the current study, both positive feelings toward the victim and negative feelings toward the offender predicted participant choices of maximum and minimum punishment for the offender. The findings highlight the importance of feelings to forensic judgments.

The results strengthen previous reports of victim-blaming in cases of NCII (Bothamley and Tully, 2018; Scott and Gavin, 2018) and add to the limited body of evidence on law enforcement victim-blaming. As NCII is a form of sexual violence, the results can be interpreted within the framework of gendered stereotypical thinking, similar to the one seen toward victims of rape. Research has showed that rape victim-blaming is predicted by sexist ideologies (Ferrão and Gonçalves, 2015) and that perceptions of rape victims are deeply influenced by myths and stereotypes as to what constitutes “real rape” and who deserves to be classified as a “real” or “genuine” victim (Randall, 2010). Among these distorted images of a “real” victim is a belief regarding the character of rape victims, whereby true victims are chaste and respectable (Farrell, 2017). Only a few studies have examined whether police officer judgments are influenced by the reputation of the victim, yet some insight is available. It was shown that police officers judged a victim purported to have a “bad” reputation as more responsible for her victimization than a victim purported to have a “good” reputation (Hine and Murphy, 2017). Similarly, analysis of real sexual assault cases and interviews with police officers showed that a victim’s moral character influenced police officer evaluations of the victim’s credibility (Campbell et al., 2015; Venema, 2016). Yet, another study showed that a complainant’s provocative dressing had no influence on her credibility or likelihood that police investigators are charging the alleged offender. However, the provocative complainant was attributed more responsibility than her counterpart who was wearing conservative clothing (Goodman-Delahunty and Graham, 2011). The current findings resemble Goodman-Delahunty and Graham (2011) in the sense that victim-blaming occurred, yet was unrelated to judgments of the offender, who was perceived as highly culpable.

NCII victims may be judged according to similar narratives of stereotypical femininity seen in the context of rape victims. Specifically, it may be argued that the mere publication of intimate images constitutes a violation of socially gendered expectations for females’ chastity. In other words, women whose intimate images were distributed may no longer be conceived as chaste or respectable, regardless of the circumstances in which their intimate images were taken or their perceived contributory role in taking the images (or lack thereof). Reality shows that many NCII victims were, in fact, photographed or video recorded by means of hidden surveillance. Regardless, according to this narrative, they may still be blamed for the mere violation of gendered social expectations. When the intimate material was generated by the victim or when the victim consented to creating it, violation of social expectations may be attributed directly to the victim and is expected to be greater. A victim who has chosen to express her sexuality may be perceived as defying social norms, resulting in greater victim-blaming.

Nevertheless, according to victim precipitation theory (e.g., Schafer, 1968, 1977), blame may be attributed to victims simply for increasing their risk of victimization. As a major premise of the theory is that victims’ behavior contributes or leads to their victimization, it is possible that participants attributed greater blame to the victim in the selfie scenario not for violating gender expectations but for her perceived contribution to the offense. However, this theory does not explain or agree with the findings on difference in feelings toward the victims in both scenarios, as well as contribution of feelings to punitive judgments.

The gender differences found in the present study are similar to the findings of studies on rape perceptions showing that men tend to attribute more blame and responsibility than women to rape victims (Furnham and Boston, 1996; Ferrão and Gonçalves, 2015; Shechory Bitton and Jaeger, 2019). Since men typically hold more sexist beliefs than women (Aosved and Long, 2006), victim-blaming among men toward victims of NCII is expected to be greater, as the current findings show. Specifically, men were victim-blaming more than women, were less empathetic toward the victim, and were expressed fewer negative feelings toward the offender. Still, it is also possible that defensive attributions and identification processes took place. Female participants could have identified with the female victim more than male participants, whereas male participants could have identified more with the male offender, leading to more lenient judgments for either of the parties.

Finally, it should be emphasized that victim-blaming did not affect participant judgments of the offender. Despite victim-blaming, police officers and students alike were generally highly convinced that the offender is blameworthy and should be brought to trial, reflecting their understanding that an offense took place, consistent with the recent criminalization of NCII. Similarly, most participants also highly advocated the maximum punishment for the offender and reported extreme negative feelings toward him, indicating their resentment toward the offender and perception of the severity of the offense. Yet some group differences were evident, as students negative feelings toward the offender were stronger than those of police officers. Additionally, unlike police officers, students were biased in their recommendations of minimum punishment, which they advised more in the selfie condition. These group differences indicate that police officers were slightly less biased than students. It seems that police officer training and experience may contribute to a more balanced approach toward NCII cases. However, as the findings indicate, these are insufficient.

## Limitations and Future Directions

Several limitations of the present study should be addressed. Future studies are advised to use random sampling of students, police officers, and lay people. Students are younger than police officers and may be more digitally savvy, and so using lay people as controls may be more suitable. Comparing the perceptions of students and lay people may further indicate whether conclusions regarding students can be generalized. It should also be noted that the study relied on self-reports, and may have thus been subjected to social desirability bias. For police officers, fearing to answer honestly on delicate topics may be particularly significant. However, this was guarded against as all the participants were assured of confidentiality and anonymity. Moreover, police officers returned their questionnaires in sealed envelopes. The results indicate biased perceptions, which suggest that officers answered the questions openly and honestly. Nevertheless, it is suggested that a measure of social desirability would be used as a control variable.

Another limitation may concern the scales used. Each of the blame and punishment variables was composed of one item, with an agreement scale ranging from very little to very high agreement. Future studies may benefit from developing or using valid scales for types of blame and punishment that include scales ranging from high disagreement to high agreement. Qualitative data may be added to enrich the understanding beyond the scope of closed scales.

In the present study, only a female victim scenario was used. Victim-blaming in sexual offenses is related to sexist and stereotypical thinking about women, the primary victims of sexual offenses. However, recent research suggests that image-based sexual abuse actually involves female and male victims at similar rates (Powell et al., 2019). This clearly challenges the dominant sexual victimization paradigm of men as perpetrators and women as victims (e.g., Shechory-Bitton and Zvi, 2020). If males are at similar risk as females to become victims of NCII, it may be argued that men should also see themselves as potential victims, which, in turn, may increase their sense of vulnerability, empathy to victims, and lessen the extent to which victim-blaming occurs. Yet, it is unclear to what degree men are aware of these statistics. It is also possible that despite awareness, the entrenched gendered social perception, whereby only women can be victims of sexual violence remains dominant. Future research using an opposite gender scenario depicting a male victim will enable us to better understand male attitudes toward NCII victims and offenders. Examining male participant knowledge of men’s risk to become victims of NCII may also contribute to understanding motivations for victim-blaming in NCII offenses. Future research should also look at the effect of same-sex NCII. Compared to heterosexuals, LGBTIQ individuals are at increased risk to become victims of a variety of crimes, and recent evidence suggests that this may also be the case for image-based sexual abuse (Henry et al., 2017, 2018b).

Future research may also benefit from looking at the perceptions of police officers from diverse cultures and backgrounds. Israeli society is still characterized by traditional gender roles (Shechory Bitton and Jaeger, 2019), and this may be particularly true with respect to some of its sub-cultures. For example, Arab, Ethiopian origin, and Former USSR origin citizens may significantly differ in their construal of gender roles. Some are more traditional and some less, and this variation may also be age related (with younger persons in traditional societies exhibiting less conservative attitudes). Different cultural characteristics may influence perceptions of offenders and victim-blaming and may be particularly relevant to perceptions of sex offenses. The literature on perceptions of NCII is limited, and future research on the issue is needed among laypersons and professional alike, assessing the various extralegal factors that may influence forensic decisions (for a review of these factors, see Goodman-Delahunty and Sporer, 2010).

Finally, NCII is a metacontextual phenomenon that should be studied in specific contexts. For example, few studies have found that experiences of victims of domestic abuse included technology-facilitated abuse, including NCII (Dimond et al., 2011; Bond, 2015). Interpersonal violence in relationships includes coercive control, where abusers use different tactics to exert power over victims, and NCII may be another route for victimization, enabling abusers to threaten, control, and abuse. Research is needed in order to establish the manner and scope in which NCII is used in such circumstances, as well as finding solutions.

## Conclusion

Israeli law states that unconsented distribution of both self-taken and stealth-taken intimate images constitutes a criminal act. The current study clearly shows that officers perceive both circumstances as criminal and the offender as highly culpable and punishable.

It was found that the magnitude of the differences is mostly greater for students, as the effect size is moderate compared with the other one that is rather small. Nevertheless, the results indicate that officers are victim-blaming and, more so, in the case when the victim’s intimate material was self-generated. Such circumstances evoked negative feelings and lower levels of empathy toward the victim. The relevance of emotions in legal contexts is emphasized in light of their contribution to participant punitive judgments.

There is a reason to suspect that the majority of NCII cases remain unreported (Bond and Tyrrell, 2018; Henry et al., 2018b), similar to other sexual offenses (Grubb and Turner, 2012; Van der Bruggen and Grubb, 2014). Negative and victim-blaming attitudes on the part of the police might increase reluctance of victims to report NCII offenses and cooperate with police investigation. They might also result in additional trauma to the victim and secondary victimization (Alderden and Ullman, 2012). As with victims of other sex offenses, victims of NCII suffer emotional trauma, loss of trust, and feelings of shame and humiliation, which render them especially vulnerable to negative experiences with the criminal justice system. Viewed more broadly, victim blaming attitudes within law enforcement promote cultural acceptance of sexual offenses and justify and normalize sexual offenses (Grubb and Turner, 2012).

The current findings indicate the importance of expanding educational and informational programs that will help change attitudes, undermine prevalent myths, and reduce the ability of both professionals and society to devalue victims. Providing meaningful statistics on NCII may also lessen negative sentiments and contribute to a more empathetic approach toward victims as well as to more objective decisions among legal professionals.

## Data Availability Statement

The datasets generated for this study are available on request to the corresponding author.

## Ethics Statement

The studies involving human participants were reviewed and approved by Ariel University Ethics committee. The patients/participants provided their written informed consent to participate in this study.

## Author Contributions

All authors contributed to planning, executing, and writing the research. All authors contributed to the article and approved the submitted version.

### Conflict of Interest

The authors declare that the research was conducted in the absence of any commercial or financial relationships that could be construed as a potential conflict of interest.
